# Treg/IL-17 Ratio and Treg Differentiation in Patients with COPD

**DOI:** 10.1371/journal.pone.0111044

**Published:** 2014-10-16

**Authors:** Yang Jin, Yong Wan, Gang Chen, Long Chen, Ming-Qiang Zhang, Li Deng, Jian-Chu Zhang, Xian-Zhi Xiong, Jian-Bao Xin

**Affiliations:** 1 Department of Respiratory and Critical Care Medicine, Key Laboratory of Pulmonary Diseases of Health Ministry, Union Hospital, Tongji Medical College, Huazhong University of Science and Technology, Wuhan, China; 2 Department of Respiratory and Critical Care Medicine, Wuhan No. 1 Hospital, Wuhan, China; Emory University, United States of America

## Abstract

**Background:**

Chronic obstructive pulmonary disease (COPD) is characterized by chronic pulmonary and systematic inflammation. An abnormal adaptive immune response leads to an imbalance between pro- and anti-inflammatory processes. T-helper (Th), T-cytotoxic (Tc) and T-regulatory (Treg) cells may play important roles in immune and inflammatory responses. This study was conducted to clarify the changes and imbalance of cytokines and T lymphocyte subsets in patients with COPD, especially during acute exacerbations (AECOPD).

**Methods:**

Twenty-three patients with stable COPD (SCOPD) and 21 patients with AECOPD were enrolled in the present study. In addition, 20 age-, sex- and weight-matched non-smoking healthy volunteers were included as controls. The serum levels of selected cytokines (TGF-β, IL-10, TNF-α, IL-17 and IL-9) were measured by enzyme-linked immunosorbent assay (ELISA) kits. Furthermore, the T lymphocyte subsets collected from peripheral blood samples were evaluated by flow cytometry after staining with anti-CD3-APC, anti-CD4-PerCP, anti-CD8- PerCP, anti-CD25-FITC and anti-FoxP3-PE monoclonal antibodies. Importantly, to remove the confounding effects of inflammatory factors, the authors introduced a concept of “inflammation adjustment” and corrected each measured value using representative inflammatory markers, such as TNF-α and IL-17.

**Results:**

Unlike the other cytokines, serum TGF-β levels were considerably higher in patients with AECOPD relative to the control group regardless of adjustment. There were no significant differences in the percentages of either CD4^+^ or CD8^+^ T cells among the three groups. Although Tregs were relatively upregulated during acute exacerbations, their capacities of generation and differentiation were far from sufficient. Finally, the authors noted that the ratios of Treg/IL-17 were similar among groups.

**Conclusions:**

These observations suggest that in patients with COPD, especially during acute exacerbations, both pro-inflammatory and anti-inflammatory reactions are strengthened, with the pro-inflammatory reactions dominating. Although the Treg/IL-17 ratios were normal, the regulatory T cells were still insufficient to suppress the accompanying increases in inflammation. All of these changes suggest a complicated mechanism of pro- and anti-inflammatory imbalance which needs to be further investigated.

## Introduction

Chronic obstructive pulmonary disease (COPD), which is primarily the result of chronic cigarette smoking and exposure to various hazardous gases and particles, is characterized by poorly reversible airflow limitation and progressive airway inflammation [1]. Although cigarette smoking is established as the main etiological factor for COPD, only a fraction of so-called “susceptible smokers” finally progress to COPD [2]. It is clear that the quantity of smoke inhaled as well as genetic elements can have coordinated effects on the development of COPD. Furthermore, the progression of COPD is essentially attributed to the dysfunction of regulatory mechanisms, including weak anti-protease [3] and anti-oxidant [4] activities, and in particular, to maladaptive immune modulation [5].

The concept of an imbalance in the Treg/Th17 relationship has been investigated quite extensively in many diseases, including COPD [6], [7], [8]. On the one hand, Tregs can serve to suppress inflammation in chronic inflammatory and autoimmune diseases via multiple pathways including directly through cell contact and/or indirectly via the secretion of TGF-β and IL-10. Tregs are capable of suppressing not only the proliferation of inflammatory cells but also the production of pro-inflammatory cytokines [9]. We have previously demonstrated that the quantity and percentage of Tregs in patients with AECOPD were significantly correlated with smoking indices and blood pH [10], indicating that Tregs may be involved in the pathogenesis of COPD. Furthermore, even within the same COPD patient, Tregs vary with the stages of the disease and the sites of sampling [11], [12], [13]. On the other hand, Th17 cells can also promote autoimmune and inflammatory diseases primarily through the generation of IL-17 and several other cytokines [14]. Most recently, studies have shown that IL-17 is elevated in patients with COPD [15]. Therefore, the authors hypothesize that anti-inflammatory Tregs are insufficiently effective for preventing lung damage, resulting in a switch in the immune response to a pro-inflammatory Th17 response [6]. However, the detailed regulatory mechanisms involved in COPD are poorly understood.

Thus, the authors aimed to investigate the cytokines and T cell subsets associated with pro- and anti-inflammatory processes in the peripheral blood of COPD patients. Importantly, to remove the confounding effects of inflammatory factors, the authors adjusted each measured value with representative inflammatory markers, such as TNF-α (an indicator of the degree of inflammation mainly resulting from innate immunity) and IL-17 (a pro-inflammatory indicator used as an internal reference). To the best of our knowledge, our study represents the first attempt to investigate the immune status of patients with COPD by means of inflammation adjustment.

## Materials and Methods

### Ethics statement

The study protocol was approved by the Institutional Review Board for human studies of Tongji Medical College, China, and written informed consent was obtained from each blood donor.

### Subjects

According to the diagnostic criteria developed by the Global Initiative for Chronic Obstructive Lung Disease (GOLD) [1], 23 patients with SCOPD and 21 patients with AECOPD were enrolled. Patients who met the criteria for COPD but had a known alternative respiratory disorder (such as bronchiectasis or severe infectious diseases) were excluded. In addition, the authors recruited 20 healthy nonsmokers (HN) as controls (clinical characteristics are listed in Table 1).

**Table 1:** Subject characteristics.

Variable	Control	SCOPD	AECOPD
Subjects	20	23	21
Age yrs	66±2.9	63±1.0	70±2.0
Male/female	18/2	20/3	20/1
Smoking history pack-yrs	-	58±3.1	60±3.4
FEV1 (% predicted)	85±2.0	45±3.0**	39±2.0**
FEV1 (% FVC)	89±1.8	50±1.6**	48±1.7**
Bronchodilation test	-	Negative	Negative

The data are presented as the mean ± SEM, unless otherwise stated. FEV1: forced expiratory volume in one second; FVC: forced vital capacity. **p<0.01 versus control group.

### Sample collection and processing

The fasting peripheral blood samples from each subject were collected into two EDTA-treated tubes and were immediately immersed in ice. One tube of the specimen was centrifuged at 1000×*g* for 15 min, and the cell-free serum was then immediately frozen at –80°C for the subsequent analysis of cytokines; the other specimen tube was used for flow cytometry, as described below.

### Determination of cytokines

The concentrations of cytokines (TGF-β, IL-10, TNF-α, IL-17 and IL-9) in serum were measured using ELISA kits according to the manufacturer’s protocols (all kits were purchased from Boster Biosciences Co., Wuhan, China). All samples were assayed in duplicate.

### Flow cytometry

Peripheral blood samples (150 µL) were incubated with APC-conjugated anti-CD3 (3.5 µL), PerCP-conjugated anti-CD4 (3.5 µL) or PerCP-conjugated anti-CD8 (3.5 µL) and FITC-conjugated anti-CD25 (3.5 µL) monoclonal antibodies (mAbs) (BD Biosciences) at 4°C for 20 min. RBC lysis buffer (1 mL) (Boster Biosciences) was then added, and the samples were incubated for another 10 min in the dark. The tubes were centrifuged at 200×*g* for 5 min. The supernatants were discarded, and the cells were washed in PBS. The cells were resuspended in Foxp3 fixation/permeabilization concentrate and diluent solutions (500 µL) (eBioscience) and incubated at room temperature in the dark for 30 min. The tubes were centrifuged at 200×*g* for 5 min, the supernatants were discarded, and the cells were then washed twice in PBS. The cells were incubated with PE-conjugated anti-FoxP3 mAb (7 µL) (eBioscience) at 4°C for 30 min. Isotype-matched mAbs were used as negative controls during each staining stage. After washing with PBS, the stained cells were analyzed using flow cytometry with a FACS Calibur (BD Biosciences) and analyzed with FCS Express V3 software (De Novo Software).

### Statistics

All of the results were analyzed using a statistical software package (Graph Pad Prism). The data are presented as the mean ± SEM, unless otherwise stated. Importantly, to remove the confounding effects of inflammatory factors on the cytokines and lymphocyte subsets, we divided each originally measured value of cytokine or flow cytometry data by the arithmetic mean of a classical inflammatory marker (TNF-α or IL-17). Differences among all groups were analyzed by one-way analysis of variance (ANOVA) followed by the SNK-q test, and P values<0.05 were considered to be statistically significant.

## Results

### Serum concentrations of different cytokines before and after adjustment for TNF-α and IL-17

**Figure 1:**
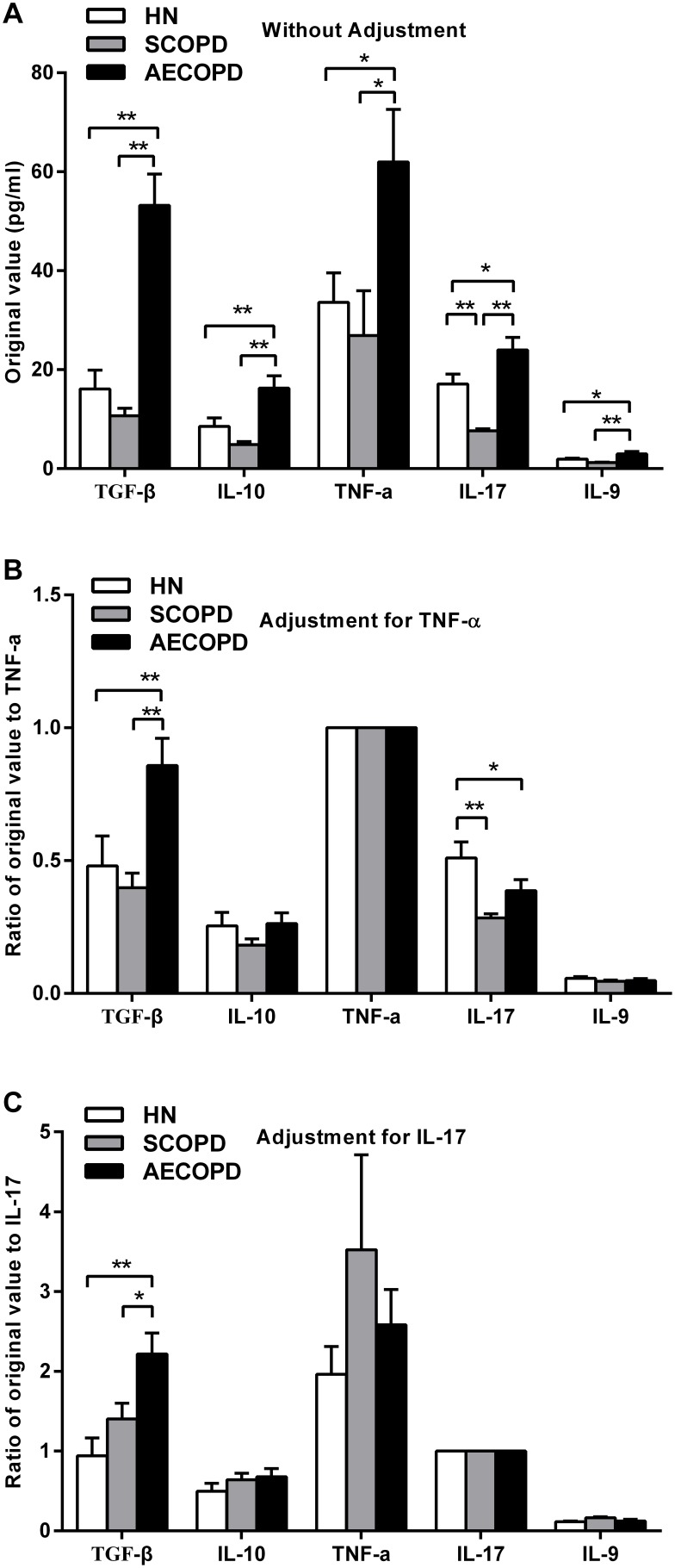
Serum concentrations of selected cytokines before and after adjustment for TNF-α and IL-17. The original values for the selected cytokines were quantitated in serum obtained from healthy nonsmokers (n = 20) and subjects with SCOPD (n = 23) and AECOPD (n = 21) by ELISA (A). To eliminate the mixed effects of inflammatory factors, the authors divided each original value by the arithmetic mean of a classical inflammatory marker, such as TNF-α (B) and IL-17 (C). The data are presented as the mean ± SEM, unless otherwise stated. *p<0.05 and **p<0.01.

To evaluate cytokine accumulation in the progression of COPD, the authors analyzed the serum concentrations of cytokines, such as TGF-β and IL-10, which are considered to be primarily anti-inflammatory, as well as pro-inflammatory cytokines, including TNF-α, IL-17 and IL-9 (Figure 1A). The serum of the AECOPD subjects had significantly higher levels of all five cytokines compared with that of the healthy nonsmokers and with that of the SCOPD patients. Interestingly, the serum concentrations of certain cytokines in the patients with SCOPD were even lower than those in the healthy controls (e.g., IL-17, 7.63±0.41 pg/ml vs 17.13±1.99 pg/ml, P<0.01).

To eliminate the mixed effects of inflammatory factors, the authors divided each originally measured value by the arithmetic means of the classical inflammatory markers TNF-α and IL-17 (Figure 1B and C). After correction, the relative serum concentrations of IL-10, TNF-α and IL-9 did not show any significant differences among groups. However, after the correction, the serum TGF-β levels were still significantly higher in the patients with AECOPD compared with those in the healthy controls and in the SCOPD patients. In contrast, both COPD groups had significantly reduced serum levels of IL-17 compared with those of the controls after adjustment for TNF-α.

### CD4^+^ and CD8^+^ T cells in peripheral blood

**Figure 2:**
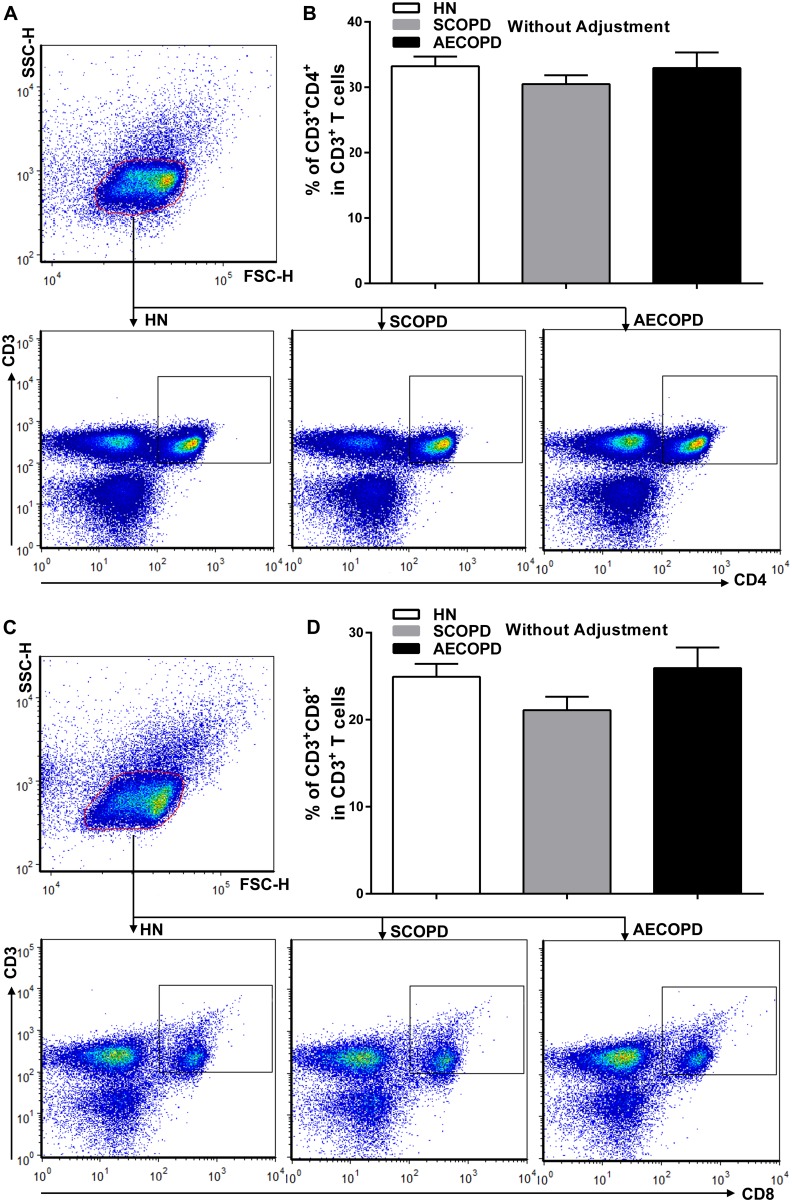
Flow cytometric analysis of CD4^+^ and CD8^+^ T cells in peripheral blood. Lymphocytes were gated on forward scatter height (FSC-H) versus side scatter height (SSC-H) plots, and representative dot plots gated on lymphocytes show CD3^+^CD4^+^ T cells (A) and CD3^+^CD8^+^ T cells (C) in the peripheral blood obtained from a single subject from each group. The collective analyses show the expression of CD4 (B) and CD8 (D) on CD3^+^ T cells from healthy nonsmokers (n = 20) and subjects with SCOPD (n = 23) and AECOPD (n = 21). The data are presented as the mean ± SEM, unless otherwise stated.

To determine the differences between the T-lymphocyte subsets between the COPD patients and the healthy nonsmokers, the authors next performed flow cytometry on mononuclear cells from peripheral blood (Figure 2). To our surprise, no significant differences were observed in the ratio of Th (CD4^+^) to T(CD3^+^) cells among the three groups (Figure 2A and B). Similarly, there were no significant differences among the groups for the ratio of Tc (CD8^+^) to T(CD3^+^) cells (Figure 2C and D).

### CD25 and FoxP3 expression on CD4^+^ T cells before and after adjustment

**Figure 3:**
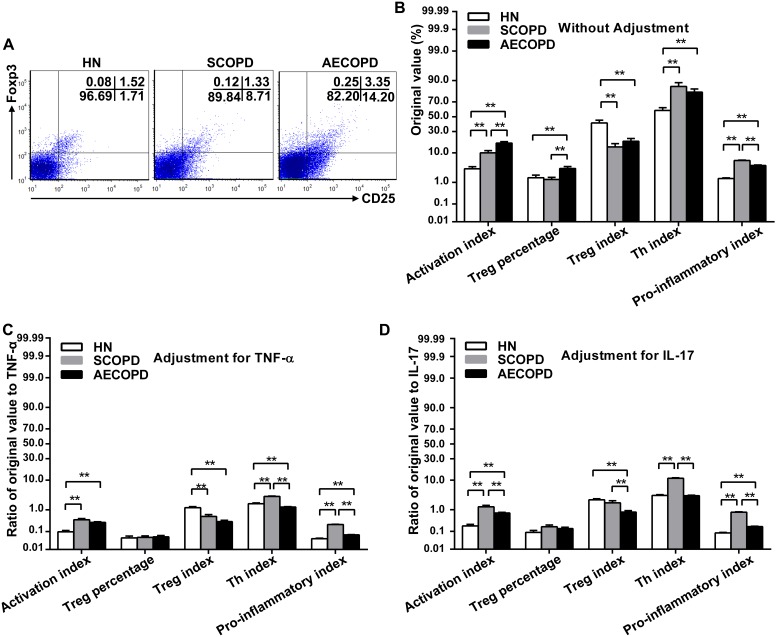
Expression of CD25 and FoxP3 on CD4^+^ T cells before and after adjustment. Representative dot plots show the expression of CD25 and FoxP3 on CD4^+^ T cells in the peripheral blood obtained from a single subject from each group (A). The original values associated with CD25 and FoxP3 expressed on CD4^+^T cells in the peripheral blood from healthy nonsmokers (n = 20) and subjects with SCOPD (n = 23) and AECOPD (n = 21) were comprehensively analyzed (B). To eliminate the mixed effects of inflammatory factors, we calculated the ratios of original values to TNF-α (C) and IL-17 (D). The data are presented as the mean ± SEM, unless otherwise stated. **p<0.01. Activation index = CD4^+^CD25^+^/CD4^+^; Treg percentage = CD4^+^CD25^+^FoxP3^+/^CD4^+^; Treg index = CD4^+^CD25^+^FoxP3^+/^CD4^+^CD25^+^; Th index = CD4^+^CD25^+^FoxP3^−^/CD4^+^CD25^+^. Pro-inflammatory index = Th index/Treg index.

Because the activation and differentiation of T cells can be characterized by the expression of CD25, the authors examined the ratio of CD4^+^CD25^+^ cells to total CD4^+^ cells as the activation index (AI). To our surprise, both COPD groups had significantly greater CD4 AI levels than those of the healthy nonsmokers (Figure 3B). Furthermore, CD4 AI level in the peripheral blood of the AECOPD subjects was greater than that of the SCOPD subjects (Figure 3B). After adjustment for the TNF-α or IL-17 levels, however, the CD4 AI levels were still higher in the COPD groups, with both p-values being smaller than 0.01 (Figure 3C and D).

Furthermore, the patients with AECOPD also had a significantly elevated percentage of CD4^+^ Tregs compared with that of the SCOPD patients and healthy controls (Figure 3B). Nevertheless, after adjustment, the Treg percentage in the AECOPD group was no longer significantly higher than that in the other two groups (Figure 3C and D). In other words, the AECOPD patients demonstrated a compensatory augmentation of Tregs in the presence of strong inflammatory signals. Notably, similar ratios of Treg/IL-17 were observed in all groups, in contrast to a previously reported imbalance of Treg/Th17 [6].

In addition, the authors defined the CD4^+^CD25^+^FoxP3^+^/CD4^+^CD25^+^ percentage as the Treg index to assess the relative numbers of differentiated CD4^+^ Tregs. Regardless of correction, both COPD groups had a decreased Treg index compared with that of healthy nonsmokers, which demonstrated a lack of differentiation ability of the Tregs (Figure 3B, C and D).

Similarly, the authors defined the ratio of CD4^+^CD25^+^FoxP3^+^ to CD4^+^CD25^+^ as the Th index. According to these definitions, the authors speculated that the Th index changed inversely with the trend of the CD4^+^ Treg index. As shown in Figure 3B, both COPD groups had significantly increased Th indices in contrast to the decreased Treg indices.

Finally, the authors viewed the ratio of the Th index to the Treg index as a pro-inflammatory index, which indicated the relative levels of the pro-inflammatory and anti-inflammatory potentials. This pro-inflammatory index was significantly higher in both COPD groups than in the controls, regardless of the manner by which the data were adjusted (Figure 3B, C and D). Surprisingly, the SCOPD patients had a significantly higher pro-inflammatory index than that observed for either the AECOPD patients or the healthy nonsmokers (Figure 3B, C and D).

### CD25 and FoxP3 expression on CD8^+^ T cells before and after adjustment

**Figure 4:**
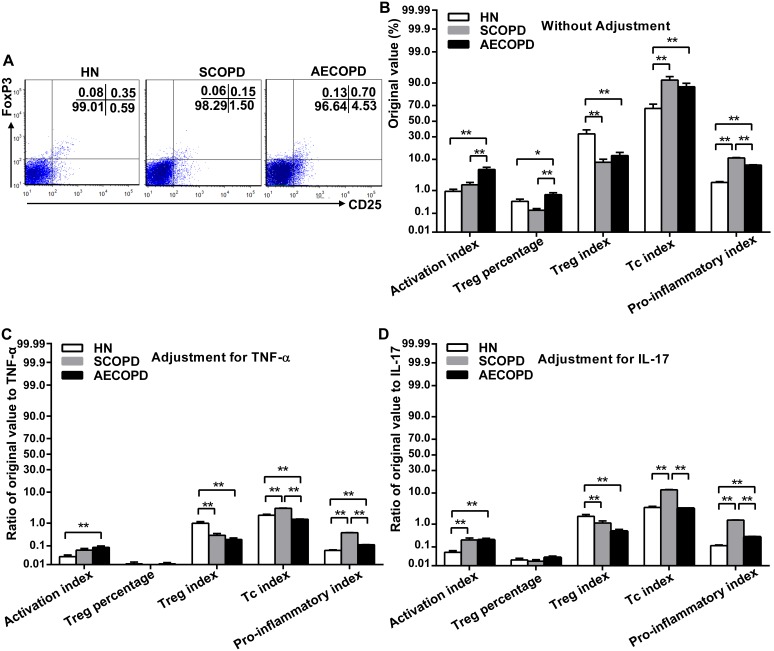
Expression of CD25 and FoxP3 on CD8^+^ T cells before and after adjustment. Representative dot plots show the expression of CD25 and FoxP3 on CD8^+^ T cells in the peripheral blood obtained from a single subject from each group (A). The original values associated with CD25 and FoxP3 expressed on CD4^+^T cells in peripheral blood from healthy nonsmokers (n = 20) and subjects with SCOPD (n = 23) and AECOPD (n = 21) were comprehensively analyzed (B). To eliminate the mixed effects of inflammatory factors, we calculated the ratios of original values to TNF-α (C) and IL-17 (D). The data are presented as the mean ± SEM, unless otherwise stated. *p<0.05 and **p<0.01. Activation index = CD8^+^CD25^+^/CD8^+^; Treg percentage = CD8^+^CD25^+^FoxP3^+^/CD8^+^; Treg index = CD8^+^CD25^+^FoxP3^+/^CD8^+^CD25^+^; Tc index = CD8^+^CD25^+^FoxP3^−^/CD8^+^CD25^+^; Pro-inflammatory index = Tc index/Treg index.

In addition, the expression of CD25 and FoxP3 on the CD8^+^ T cells was determined to evaluate whether the results were consistent with those obtained for the CD4^+^ T cells (Figure 4). Although the CD8^+^ T cells had relatively low levels of CD25 and FoxP3, the observed alterations in the subset distribution among the CD8^+^ T cells were similar to those of the CD4^+^ T cells. Notably, the CD8^+^ AI in the patients with AECOPD was enhanced 3.18-fold relative to that of the SCOPD subjects (5.24±0.89% vs 1.65±0.35%, P<0.01) (Figure 4B). However, the CD4^+^ AI of the AECOPD group was only 1.74 times greater than that of the SCOPD group (17.50±1.32% vs 10.03±1.42%, P<0.01) (Figure 3B). These results indicate that the increased reactivity of CD8^+^ T cells was involved in acute exacerbations.

## Discussion

There are conflicting reports regarding various changes of cytokine levels in COPD, and these systemic and local changes may vary continuously as a reflection of disease severity. In general, inflammation is characterized by the upregulation of both pro-inflammatory and anti-inflammatory cytokines, including different cytokine inhibitors and their soluble receptors [16]. In this study, our results demonstrated that the concentrations of cytokines such as TGF-β and IL-10, which have predominantly anti-inflammatory effects, as well as pro-inflammatory cytokines, such as TNF-α, IL-9 and IL-17, were significantly higher in serum of the AECOPD patients compared with those in the SCOPD patients and healthy controls. Interestingly, the serum of the SCOPD subjects had even lower levels of certain cytokines than that observed in the serum of the controls, which suggested a partial remission of the inflammatory condition during the stable phase. Unfortunately, there were no significant differences in the levels of TNF-α, IL-9 and IL-10 among the three groups after adjustment using the representative inflammatory markers TNF-α or IL-17. Therefore, the authors focused only on TGF-β and IL-17 which still remained significant differences among the groups remained even after adjustment.

High serum TGF-β levels are found in patients with COPD [17], [18], and TGF-β, as a critical factor of COPD, is expected to become a treatment target of the disease [19]. In the present study, the circulating levels of TGF-β in patients with AECOPD were significantly greater than those observed in the other groups, both before and after adjustment. What can we speculate from such high levels of TGF-β in the peripheral blood of AECOPD patients? Some investigators have demonstrated that TGF-β can serve as a signal to suppress the immune response and inflammation [20]. However, it is not clear in the present study why such a high concentration of TGF-β failed to adequately suppress chronic inflammation.

One possibility is that the simple normalization to the TNF-α or IL-17 concentrations provided an incomplete view of the overall pro/anti-inflammatory state. Numerous pro-inflammatory and anti-inflammatory cytokines may interact in the complex networks associated with immune disorders. Alternatively, recent reports have suggested that the TGF-β pseudoreceptor BMP- and activin-membrane-bound inhibitor (BAMBI) can negatively regulate the TGF-β signal by competing with other TGF-β receptors for binding to TGF-β [21]. The expression of BAMBI could be influenced by multiple factors; for example, TGF-β could upregulate BAMBI, which would result in decreased effectiveness of TGF-β. It has been reported that BAMBI is strongly expressed in the lungs of patients with COPD and is regulated by hemophilic influenza [22]. To the best of our knowledge, no previous studies have focused on the function of BAMBI in lymphocytes. Our preliminary experiment (unpublished data) showed that lymphocytes express BAMBI and that BAMBI could be regulated by cigarette smoking extract (CSE). Therefore, further work is needed to elucidate the abnormal TGF-β signaling pathway in patients with COPD.

IL-17 is acknowledged to be a pro-inflammatory cytokine that is mainly produced by Th17 or Tc17 cells [23]. This cytokine can facilitate the proliferation of T cells and the expression of various inflammatory mediators while, at the same time, enhancing neutrophil chemotaxis. A study of mouse pulmonary epithelial cells revealed that the overexpression of IL-17A induced COPD-like pulmonary inflammation [24]. It has also been reported that IL-17 and Th17 were increased in patients with COPD [15], [25], but these reports did not include the information required to evaluate whether this increase was consistent with the degree of inflammation. Our current study demonstrates that although IL-17 levels in the peripheral blood derived from patients with AECOPD were relatively increased, these levels unexpectedly became significantly lower than that in the controls when the authors eliminated the effects of TNF-α. The factors underlying the limited production of IL-17 are therefore of interest. Generally, in patients with AECOPD, the higher level of TGF-β coupled with the increase in IL-6 [26], [27] would intensely promote the generation of IL-17 and Th17 cells [28], [29]. Whether the high levels of TGF-β suppressed the production of IL-17 or the high expression of BAMBI prevented TGF-β and IL-6 from converting naïve CD4^+^ T cells into Th17 cells remains to be explored in the future. We believe that both internal and external environments can affect individuals and that the combination of these effects results in the refractory chronic inflammation.

As for the T cell subsets, considerable effort has been directed toward the identification of their specific impact on patients with COPD. From the results obtained so far, it seems that studies have mainly concentrated on the influence of CD8^+^ T cells [30], [31], [32], but the contributions of these cells to the inflammatory pathway are less clear. Animal studies have revealed a crucial role for these cells, as indicated by the fact that CD8^+^ T cell-deficient (CD8−/−) mice did not develop emphysema during long-term exposure to cigarette smoke [32]. However, our present data found no significant differences in the proportion of either CD4^+^ or CD8^+^ T cells between the COPD groups and healthy controls.

The activation and differentiation of T cells can be characterized by the expression of CD25. The authors have noted that the CD4 AI in the cells of the peripheral blood from each of the COPD groups was higher than that of the healthy controls. Furthermore, the AECOPD patients had significantly greater CD4 AI than the SCOPD patients. After adjustment for TNF-α or IL-17, however, the CD4 AI was still significantly greater in both COPD groups. It has been shown that CD25, which is vitally important to the delivery of IL-2 signals to Tregs, is strongly expressed on CD4^+^CD25^+^FoxP3^+^ Tregs and activated T cells [33]. Enhanced levels of AI suggest that there was extensive inflammation in the patients with COPD, especially the AECOPD patients, which could irreversibly alter immune homeostasis.

It is still controversial whether Tregs are increased in COPD [10], [11], [12]. Our present study demonstrated that patients with AECOPD also had a significantly elevated percentage of CD4^+^ Tregs compared with those of the SCOPD patients and healthy controls. Nevertheless, after correction for TNF-α or IL-17, the Treg percentage did not show any significant differences between the patients and controls, indicating that both TNF-α and IL-17 contributed to the differentiation of Tregs to some extent during acute exacerbations. The authors hypothesize that Tregs proliferate in a compensatory manner under the stimulation of pro-inflammatory cytokines, although the levels of proliferation are not enough to govern the burst of inflammation during an acute exacerbation. Notably, the similar ratios of Tregs/IL-17 in all groups contrasted with the reports by other investigators [6] that demonstrated the existence of a Treg/Th17 imbalance in patients with COPD.

Recently, researchers have observed that fresh pulmonary Tregs prevented the proliferation of Th17 cells but not their capacity to secrete IL-17, although there was no correlation between Tregs and IL-17-producing cells [7]. Overall, the different experimental conditions used in the literature make it difficult to draw consistent conclusions regarding the relationship between Tregs and Th17 cells. However, our data demonstrated that the CD4^+^ Treg index (namely the differentiation rate for the Tregs) was lower in both COPD groups than in the controls regardless of adjustment [34]. Combining previous concepts developed by others [6], [12] with the results of this study, the authors hypothesize that although acute inflammation may be the main stimulator that promotes the development of Tregs, the anti-inflammatory properties could not provide essential protection to the body from an overactivated immune response. Given that TGF-β acts as a necessary promoter of Tregs [20], it was puzzling that the high TGF-β levels did not induce more Tregs. Unfortunately, the authors could not further determine the mechanism for the deficiency of Tregs in the context of the extensive increase of TGF-β. As noted above, it is possible that BAMBI was partially involved.

Similarly, to describe pro-inflammatory power, the authors defined the ratio of CD4^+^CD25^+^FoxP3^−/^CD4^+^CD25^+^ as the Th index. According to this definition, we speculate that the Th index changed inversely with the trend of the CD4^+^ Treg index. Obviously, both COPD groups had significantly greater Th indices in contrast to the decreased Treg indices in the control group.

In addition, the authors viewed the ratio of the Th index to the Treg index as a pro-inflammatory index, which indicated the balance between the pro-inflammatory and anti-inflammatory factors. Independent of the method used to adjust the data, the pro-inflammatory indices were significantly higher in both COPD groups than those in the controls, which suggested activated inflammation in COPD. Because the healthy controls had no smoking history, the authors were unable to confirm whether the discrepancy was directly correlated with disease or was simply linked directly to the number of pack-years. However, the SCOPD patients had a significantly greater pro-inflammatory index than that of either the AECOPD patients or the healthy nonsmokers. These complex results implied there was an activated inflammatory response and fewer compensatory Treg proliferation during the stable stage.

Our latest research reported that after treatment with a muscarinic cholinergic receptor antagonist (Tiotropium), the CD8^+^ Treg index was significantly increased in patients with SCOPD [34]. Thus further experiments were performed to determine whether the expression of CD25 and FoxP3 on CD8^+^ T cells was consistent with the effects observed in CD4^+^ T cells. Although CD8^+^ T cells have relatively low levels of CD25 and FoxP3, the alterations in the subset distribution among the CD8^+^ T cells were similar to those of the CD4 T cells. The authors noted that when compared with SCOPD group, the CD8 AI in the patients with AECOPD displayed a greater enhancement than the CD4 AI (3.18-fold vs 1.74-fold). To some extent, the observation that overactivated CD8^+^ T cells are involved in acute exacerbations was consistent with previous research [30], [31].

In a healthy individual, CD4 and CD8 Tregs appear to expand and contract in parallel with conventional T cells during a primary immune response and exceed the quantity of conventional T-cell subsets at the peak of the immune response [35]. Once the antigen is cleared, the Treg pool in vivo may control an ongoing primary immune response to resolve immune activity [35]. In contrast, COPD patients fail to carry out the normal regulation described above. In summary, our study reveals that in patients with SCOPD, especially in those with AECOPD, the pro-inflammatory responses are enhanced, but only limited anti-inflammatory responses occur, and these systemic alterations may have a complicated effect on local pulmonary changes. A better understanding of these immunological mechanisms will create opportunities for the development of new therapeutic strategies in disease states.
